# Parents’ Feelings, Coping Strategies and Sense of Parental Self-Efficacy When Dealing With Children’s Victimization Experiences

**DOI:** 10.3389/fpsyt.2019.00700

**Published:** 2019-10-04

**Authors:** Joy Benatov

**Affiliations:** ^1^Department of Psychology, College of Management Academic Studies, Rishon Lezion, Israel; ^2^Department of Special Education, University of Haifa, Haifa, Israel

**Keywords:** parental self-efficacy, bullying, coping, victimization, emotions

## Abstract

**Background:** Accumulating evidence strongly suggests that bullying victimization poses a major risk for children’s and adolescents’ socioemotional development. Despite the key role parents play in their child’s ability to cope with bullying, very few studies have focused on parents’ reactions to their children’s victimization. The current study examined parents’ feelings, coping strategies, and sense of parental self-efficacy subsequent to their children’s victimization.

**Methods:** The sample was composed of 217 parents of children aged 7 to 18 years who had been victims of bullying. Parents were requested to fill in a self-report survey measuring their responses to their child’s bullying victimization in the last 12 months, the feelings they experienced, the coping strategies they implemented, and their sense of parental self-efficacy in dealing with the situation.

**Results:** Parents of victimized children experienced notable emotional distress and an array of complex emotions. A unique pattern of associations was revealed between feelings and coping tactics. Specifically, feelings of guilt were predictive of parents adopting avoidance and self-blame strategies and negatively associated with providing support to the child. Parents’ feelings of sadness positively predicted coping by providing child support and negatively linked to avoidance coping. Anger was predictive of retaliative coping, whereas worry contributed to child restrictions. Providing support to the child and retaliation positively contributed to parental self-efficacy in dealing with the victimization events, whereas seeking social support was negatively associated with parents’ sense of efficacy.

**Discussion:** It is suggested that bullying prevention efforts should include parents and address the complex feelings they experience, especially feelings of guilt and anger, which were found to contribute to a maladaptive coping reaction.

## Coping With Bullying

A recent national poll conducted in the United States revealed that parents’ top health concern is bullying/cyberbullying (1). This is understandable in light of accumulating evidence indicating that bullying victimization is a major risk to children’s and adolescents’ socioemotional development [e.g., (2)]. Being bullied is an intense interpersonal stressor that serves as a catalyst for the emergence of psychosocial difficulties (3). Parental support can buffer the adverse effects of bullying to some extent (4). However, children’s bullying experiences are a major source of stress for parents as well and have a negative impact on parents’ well-being (5, 6). This underscores the crucial need to better understand parents’ psychological processes when coping with their child’s peer-victimization events.

The transactional model of stress and coping (7) suggests that when people experience an event, they evaluate whether it is threatening to their well-being (primary appraisal) and whether they have the resources to cope with it (secondary appraisal). The model points to the importance of emotion in the coping process (7, 8). Primary appraisal generates an emotional response that can vary in intensity and valence. Thereafter, coping strategies are engaged to alter the person-environment relationship by adopting strategies to either regulate distressing emotions or impact the problem causing the distress (9). Coping strategies are diverse and context-dependent (for a review, see 10). In the context of bullying victimization, the best documented children’s coping strategies include turning to an adult or peer for help, avoiding the bully or ignoring the situation as a whole, retaliating, or fighting back (11). Hunter and Boyle (12) and Hunter and Borg (13) found supporting evidence that emotions are linked to the coping strategy implemented by school-aged children victims of bullying. For example, they found that feelings of anger and self-pity predicted seeking support from peers and adults, whereas feeling helpless or indifferent predicted doing nothing as a coping tactic (13).

Despite the key role played by parents in children’s ability to cope with bullying, relatively few studies have focused on the parents’ emotional well-being or ability to cope (14). Rather, most works on bullying that have included the parents’ perspective have dealt with the parents’ definitions of bullying (6, 15), parental awareness of their child’s involvement in bullying (16, 17, 6, 18) or parents’ attitudes toward bullying and intervention efforts (19). A few qualitative studies have explored parents’ emotional experiences when their children were bullied and have revealed that parents experience worry, concern, anger, guilt, frustration, disappointment, and a sense of powerlessness (5, 6, 20).

The coping strategies parents recommend to bullied children include first turning to an adult for help, followed by ignoring the child who bullied them, or either retaliating or promoting prosocial behaviors (21, 6, 22). The coping reactions parents themselves implemented include obtaining antibullying information, consulting with others, contacting the school, and (rarely) contacting the parents of the bully (6, 21, 22). Interestingly, one study found that parents’ experiences of being bullied during childhood were associated with parents’ current views and concerns about their children’s school bullying (21).

## Parental Self-Efficacy

Parental self-efficacy refers to caregivers’ beliefs about their ability to parent successfully (23). It is an extension of the more general cognitive construct of personal self-efficacy first defined by Bandura (24). Self-efficacy is a higher-order construct that can have reciprocal associations with experiences of stress and coping efforts (25). A national poll conducted in Australia in 2018 indicated that half of all parents felt they needed more information and skills on how to deal effectively with bullying. Many parents often felt helpless when their children were bullied (26). These parental disclosures are suggestive that some parents feel their parental self-efficacy is limited when it comes to dealing with bullying. In order for parents to effectively support their child during a crisis involving bullying, they need to feel able to do so. Parental self-efficacy has been related to parental competence, parental psychological functioning, and child socioemotional adjustment (for a review, see 23). A recent study found that parental self-efficacy specifically with regard to bullying, but not general parental self-efficacy, was associated with children’s bullying and victimization behaviors (27). This points to the need to explore the precursors contributing to parental self-efficacy when dealing with bullying.

## The Current Study

The current study explored how parents’ emotional reactions contribute to their preferential coping strategies when dealing with their children’s victimization incidents and the extent to which parents’ emotions and coping strategies are associated with parental self-efficacy in the context of bullying victimization. We hypothesized that children’s bullying experiences are likely to elicit diverse feelings in parents that differ in intensity and valence. Specifically, we predicted that parents would report feelings of anger, worry, guilt, and sadness to varying extents.

Different feelings would predict different parental coping strategies. Specifically, it was posited that anger would contribute to the extent to which parents adopted retaliative coping tactics, whereas feeling worried and sad would predict utilizing child support and seeking social support and information. Feelings of guilt were expected to predict adopting a self-blame stance.

It was further assumed that the feelings parents experience and coping strategies they adopt would significantly contribute to parental self-efficacy when dealing with children’s peer victimization. Furthermore, adaptive coping strategies (child support, seeking social support and information) should positively contribute to parents’ sense of self-efficacy in dealing with bullying incidents, whereas maladaptive coping strategies (retaliation, avoidance, self-blame, child restriction) should be linked to lower parental self-efficacy. No specific predictions were made with regard to the contribution of parents’ feelings to parental self-efficacy because this is a new area of inquiry.

## Methods

### Participants

The sample was composed of 217 parents aged 24 to 59 years (mean = 39.83, SD = 6.63 years), of whom most were mothers (n = 170 [78.3%]). Their children ranged in age from 7 to 18 years (mean = 11.02, SD = 2.57 years); 59% (n = 128) were boys, and 37.4% (n = 80) were the eldest. Parents reported having on average three children; 85% of these parents lived together. Most parents, 59% (n = 128), were Jewish, 24% (n = 52) were Muslim, 24% (n = 29) were Druze, 3.7% (n = 8) were Christian, and 13.4% (n = 29) indicated other religious-ethnic origins.

### Procedure

After approval by the ethics committee at the University of Haifa, several schools in the northern district of Israel were approached. The aim of the study was presented, and permission from the schools’ administration was requested to present the study to the students’ parents. Five schools were willing to engage in the study. Parents were approached before or after school events (PTA meetings, school ceremonies) and filled in the questionnaire manually or if they preferred by email. Two hundred seventeen parents reported that their child had been victimized to some extent in the last 12 months.

Prior to running the study, a small-scale pilot study, which included semistructured interviews with eight parents whose children had been victimized in the past year, was conducted. Parents’ age was between 40 and 52 years; they volunteered for the study *via* announcements about the study posted in different online forums. In the interviews, parents talked about their ordeal, and if they did not bring it up, they were asked as to their feelings and how they dealt with the events. Afterward, they completed the questioners and provided feedback on them. The results from the pilot study were not published and only served the purpose of gaining a better understanding of the researched phenomena.

### Measures

#### Bullying Victimization

Bullying victimization was measured using six questions from the Global School-Based Student Health Survey (28) on various types of victimization (physical, verbal, relational, cyber) that occurred in the last 12 months. Parents were asked to rate each question on a 1 (never) to 4 (most days) scale indicating the frequency of the victimization. The Cronbach alpha in the current sample was.82.

#### Parents’ Emotional Experience

The feelings parents experienced after their children were bullied were measured using parents’ self-reports of their subjective emotional experience. Parents were asked to what extent (1 [not at all] to 5 [very much]) words listed in the questionnaire described the emotions they experienced when finding out about the victimization incidents. The following words were presented: frustrated, preoccupied, indifferent, embarrassed, offended, angry, guilty, sad, worried, proud, and irritated. The words were based on previous findings (5, 6, 20) on the emotions parents felt after hearing about bullying incidents indifferent and proud were added as irrelevant feelings to verify authentic responses. A similar questionnaire has been used in studies on children’s emotions when bullied (29).

#### Parents’ Strategies to Cope With Bullying Victimization

This scale was constructed for this study based on the Questionnaire of Parental Coping Strategies for Bullied Children (22) but was extended to include additional coping strategies. The expansion of this measure was theoretically based on the literature of coping and empirically on scales for victimized children’s coping and teacher’s management of bullying developed by Kochenderfer-Ladd & Skinner (30), Kochenderfer-Ladd (29), and . Two additional coping strategies (self-blame, child restriction) parents discussed in qualitative studies were included as well (5, 6, 20). Prior to running the study, the scale was used in a small-scale pilot study. In total, the scale was composed of 18 items. Parents rated on a 1-point (not at all) to 5-point (very much) scale the extent to which they had implemented the described actions after finding out about their child’s victimization experiences in the past year. Factor analysis using varimax rotation identified six factors as follows: (1) providing support and advice to the child included four items (e.g., I offered my child support and encouragement); (2) parents’ search for social support and information about bullying included four items (e.g., I turned to a friend for advice or support); (3) retaliation included three items (e.g., I threatened the other children involved); (4) avoidance included three items (e.g., I ignored the matter); (5) self-blame included two items (e.g., I blamed myself); (6) child restriction included two items (e.g., I imposed sanctions or prohibitions on my child). **Table 1** presents the factor loading values from the principal component analysis for this questionnaire. The Cronbach α’s were .94, .79, .71, .94, .93, and .68 for each subscale, respectively.

**Table 1:** Principal component analysis for Parents Copying Strategies with Victimization Questionnaire.

Parents Copying Strategies items			Factor			
	I	II	III	IV	V	VI
**Provide Support & Advise to Child (륜α = .94)**						
I offered my child possible solutions.	.92					
I offered my child support and encouragement.	.89					
I heard out my child’s side of the story.	.88					
I proposed to my child ways to prevent such situations in the future.	.88					
**Social support & Infornation (륜α = .79)**						
I turned to professional held for advice or support.		.82				
I turned to a friend for advice or support.		.79				
I consulted with the school staff (e.g. school counselor, homeroom teacher).		.66				
I read and learn about bullying.		.63				
**Retaliation (륜α = .71)**						
I threatened the school.			.83			
I complained about the school to authorities outside the school.			.86			
I threatened the other children involved.			.77			
**Avoidance (륜α = .74)**						
I told myself this is how kids are.				.88		
I told myself it’s no big deal.				.86		
I ignored the matter.				.64		
**Self-blame (륜α = .93)**						
I blamed myself.					.92	
I was angry with myself.					.90	
**Child Restriction (륜α = .68)**						
I laid sanctions or prohibitions on my child.						.81
I prevented my child from further meeting with the other children involved.						.77

#### Parental Efficacy

Parents were asked to rate how confident they were in their ability to deal with their child’s victimization. Responses were made on a 1- (not at all) to 5-point (very much) scale. This item was adapted to the context of victimization from a more general parental-efficacy scale (31).

#### Parents’ Victimization History

Parents’ victimization history was assessed using a single item “Were you a victim of bullying during childhood?” Responses were made on a 1- (never) to 5-point (very frequently) scale.

### Data Analysis

Descriptive statistics were calculated for all variables. Then, hierarchical linear regression models were conducted to predict each of the coping strategies parents used. Demographic variables, parents’ victimization history, child’s victimization levels, and the different feelings parents experienced when finding out about their child’s victimization were entered as predicting variables. Family-wise Bonferroni correction was applied, and significance level was set at .008. Finally, a hierarchical linear regression model predicting sense of parental efficacy in dealing with victimization was conducted. In this model, demographic variables, parents’ victimization history, child’s victimization levels, parents’ feelings, and coping strategies were included as predictive variables.

## Results

### Feelings Parents Experienced Following Bullying Victimization of Their Child

Parents reported feeling preoccupied (mean = 3.43, SD = 1.22), frustrated (mean = 3.12, SD = 1.28), and irritated (mean = 3.01, SD = 1.37) to a high to medium extent after finding out about their child’s victimization experiences. They reported feeling a medium to low degree of anger (mean = 2.96, SD = 1.28), offense (mean = 2.60, SD = 1.29), worry (mean = 2.74, SD = 1.27), and sadness (mean = 2.74, SD = 1.26). A very low extent of feelings of guilt (mean = 1.90, SD = 1.12) or embarrassment (mean = 1.80, SD = 1.10) was reported Victimization type and degree as reported by parents is presented in **Table 2**.

**Table 2:** Victimization levels and types as reported by the parents (N = 217).

Type of bullying victimization	M	(SE)	Percentage of victims experiencing victimization more than once a week
Verbal (0-3)	.84	(.60)	33.3%
Physical (0-3)	.48	(.61)	16.6%
Relational (0-3)	.57	(.70)	9.7%
Cyber (0-3)	.39	(.62)	4.2%

### Coping Strategies Implemented by Parents Following Bullying Victimization of Their Child

The most common coping strategy used by parents was providing support to their child (mean = 4.07, SD = 0.89), in that 85% of the parents reported applying this tactic to a medium to high extent. The second coping strategy was seeking social support and information (mean = 2.63, SD = 1.02); 48% of the parents reported applying this tactic to a medium to high extent. The remaining of the coping strategies were less frequently used. On average parents, enforced a low degree of child restrictions (mean = 1.89, SD = 0.94), although 64% imposed some after victimization incidents. Fifty percent of the parents reported feeling self-blame to some extent, but on average self-blame was low (mean = 1.74, SD = 0.97). Parents tended not to avoid or ignore bullying (mean = 1.59, SD = 0.69). Finally, parental retaliation strategies were infrequent (mean = 1.30, SD = 0.67); only 27% stated they adopted such tactics.

Hierarchical regression models predicting parents’ coping strategies revealed parents’ feelings to be clearly associated with the coping strategies they tended to use (**Tables 3** and **4**). Specifically, parents’ feelings of guilt were negatively associated with providing support to their child, whereas sadness and preoccupation, although not significant, tended to be positively associated to child support. Surprisingly, the child’s victimization level was negatively associated with child support. Overall, the model predicting child support tactics was significant (*F* = 5.23; *df* = 17,210; *p* < .001) and predicted 26% of the variance.

**Table 3:** Hierarchical regression model predicting parents’ adaptive coping strategies following bullying victimization incidents.

Variable	Child Support			Social Support		
	B	SE	β	B	SE	β
Parent’s age	–.01	.01	–.08	–.01	.01	–.09
Parent’s gender	–.06	.15	–.03	–.01	.15	–.01
Parent’s religiosity	–.01	.08	–.01	.07	.08	.05
Parents living together	–.18	.17	–.07	.03	.17	.01
Parents victimization	–.12	.08	–.10	–.06	.08	–.04
Child’s age	.02	.03	.04	.01	.03	.03
Child’s gender	.10	.12	.05	.02	.12	.01
Child birth order	–.06	.06	–.08	–.13	.06	–.16^+^
Child’s victimization	–.09	.02	–.30**	.01	.02	.03
Frustrated	.03	.08	.05	.19	.07	.23^+^
Preoccupied	.17	.08	.24^+^	.06	.08	.07
Offended	–.06	.06	–.09	.12	.06	.15^+^
Worried	.05	.06	.07	–.02	.06	–.03
Irritated	.11	.07	.17	.01	.06	.01
Angry	.05	.06	.07	.06	.06	.07
Sad	.15	.06	.21^+^	.12	.06	.15
Guilty	–.18	.07	**–.23**	.13	.07	.14
	F _17,210_ = 5.23** Adj R^2^ = .26			F = _17,210_ 10.52** Adj R^2^ = .44		
^+^ *p* < .05 * *p* < .008 ** *p* < .001						

**Table 4:** Hierarchical regression model predicting parents’ adaptive coping strategies following bullying victimization incidents.

Variable	Retaliation			Avoidance			Self-blame			Child Restriction		
	B	SE	β	B	SE	β	B	SE	β	B	SE	β
Parent’s age	–.07	.01	–.07	–.01	.01	–.07	–.00	.01	–.02	–.01	.01	–.02
Parent’s gender	–.12	.12	–.08	–.07	.12	–.04	–.20	.12	–.09	.21	.15	.09
Parent’s religiosity	–.03	.06	–.04	–.03	.07	–.03	.16	.06	.12^+^	.10	.08	.08
Parents living together	.11	.13	.06	.43	.14	.21*	.02	.12	.01	.30	.15	.12^+^
Parents victimization	–.08	.07	–.10	.13	.07	.14	–.02	.07	–.01	.13	.09	.10
Child’s age	.01	.02	.05	.01	.02	.04	.01	.02	.03	–.02	.03	–.06
Child’s gender	.03	.10	.02	–.16	.10	–.12	.16	.10	.08	–.54	.13	–.28**
Child birth order	.01	.04	.02	.04	.05	.07	–.05	.05	–.07	–.14	.06	–.17**^+^**
Child’s victimization	.06	.02	.29**	.06	.02	.24*	.05	.02	.14^+^	.01	.02	.04
Frustrated	.01	.06	.02	–.04	.06	–.07	–.08	.06	–.10	.06	.08	.07
Preoccupied	–.02	.06	–.03	–.06	.06	–.11	.10	.07	.12	–.16	.08	–.15
Offended	–.07	.04	–.14	.05	.05	.10	–.03	.05	–.04	–.03	.06	–.05
Worried	.01	.04	.01	.03	.05	.06	.01	.05	.01	.23	.05	.31**
Irritated	.05	.05	.11	–.07	.05	–.13	.10	.05	.14	.01	.07	.01
Angry	.14	.05	.29**	–.01	.05	–.01	–.02	.05	–.03	.12	.07	.16
Sad	–.08	.05	–.16	–.15	.05	–.28*	–.05	.05	–.06	–.05	.07	–.07
Guilty	–.01	.05	–.02	.14	.05	.23*	.62	.05	.70**	.10	.07	.12
	F = _17, 210_ 2.54** Adj R^2^ = .11			F _17, 210_ = 3.23** Adj R^2^ = .15			F = _17, 210_ 17.91*** Adj R^2^ = .58			F_17, 210_ = 5.65*** Adj R^2^ = .27		

^+^p < .05 * p < .008 ** p < .001 *** p < 0.001.

In terms of seeking out social support and information, the prediction model was significant (*F* = 10.52; *df* = 17,210; *p* < .001) and accounted for 44% of the variance. However, none of the predicting variables reached the Bonfferoni adjusted significance level individually. Parents’ feelings of frustration and offense tended to be positively associated with seeking social support, but this trend did not reach the required significance level.

Parents’ feelings of anger and child’s level of victimization were positively associated with retaliation. Overall, the model predicting retaliation was significant (*F* = 2.54; *df* = 17,210; *p* < .001) and predicted 11% of the variance.

Avoidance was significantly predicted by parents’ feelings of guilt and child’s victimization levels. Feelings of sadness were negatively associated with avoidant tactics. Overall, the model predicting avoidant coping was significant (*F* = 3.23; *df* = 17,210; *p* < .001) and predicted 15% of the variance.

Not surprisingly, feelings of guilt predicted parents’ self-blame. Feelings of guilt alone accounted for 58% of the variance and were the sole significant predictor (*F* = 3.06; *df* = 17,210; *p* < .001) in this model.

Parents’ worry was predictive of child restriction strategies. Boy victims also positively predicted child restriction strategies. Overall, this model was significant (*F* = 5.62; *df* = 17,210; *p* < .001) and accounted for 27% of the variance.

### Parental Self-Efficacy in Dealing With Bullying Victimization Incidents

The parental coping strategies of child support and retaliation positively contributed to parental self-efficacy in dealing with victimization incidents (**Table 5**). By contrast, social support seeking was negatively associated with parental self-efficacy. The predictive model was significant (*F* = 4.43; *df* = 23,210; *p* < .001) and accounted for 29% of the variance. Parents’ feelings did not significantly contribute to parental efficacy above and beyond coping tactics.

**Table 5:** Hierarchical regression model predicting parental self-efficacy following bullying victimization incidents.

	Parental self-efficacy		
Variable	B	SE	β
			
Parent’s age	–.01	.01	–.01
Parent’s gender	.29	.17	.11
Parent’s religiosity	–.07	.09	–.05
Parents living together	–.08	.19	–.03
Parents victimization history	.15	.09	.11
Child’s age	.02	.03	.06
Child’s gender	–.13	.15	–.06
Child birth order	–.04	.07	–.06
Child’s victimization	–.04	.03	–.11
Frustrated	–.01	.09	–.01
Preoccupied	–.01	.09	–.02
Offended	.10	.07	.13
Worried	.07	.07	.09
Irritated	–.10	.07	–.14
Angry	.08	.07	.11
Sad	.10	.08	.12
Guilty	–.01	.10	–.01
Child support	.55	.08	.48***
Social support	–.22	.08	–.21*
Retaliation	.37	.10	.23**
Avoidance	.01	.10	.01
Self-blame	–.04	.10	–.04
Child restriction	–.08	.08	–.09
	F _23,210_ = 4.73*** Adj R^2^ = .29		

**p < .05 ** p < .01 *** p < .001

## Discussion

The main goal of the present study was to explore how parents’ feelings subsequent to child victimization contributed to their preference for coping strategies and how these feelings and strategies contributed to parents’ sense of efficacy in dealing with bullying victimization.

The hypotheses were partially confirmed. The findings showed that parents experience a diverse array of feelings after learning that their child has been victimized. These included a substantial amount of preoccupation, frustration, irritation, anger, sadness, and offense. This is consistent with previous qualitative studies in which parents expressed feelings such as being powerless, angry, and worried (6, 14, 32). The current study constitutes the first quantitative attempt to measure parents’ feelings and explore their contribution to parents’ coping choices and sense of efficacy in bullying situations. An important finding in this regard was the role of guilt. A recent study by Hale et al. (32) found guilt to be a main theme expressed by parents of bullying victims. Parents tend to take on the role of protector of their child, and assessment of their actions often led to self-blame. In some cases, parents felt responsible for their child’s situation; as one mother said, “What have I done wrong or what haven’t I done? …we should have stayed in London … what if I hadn’t got divorced?” (32). The current findings go a step further and show that parents’ feelings of guilt may play a maladaptive role in parents’ coping choices since these feelings were predictive of adopting avoidance and self-blame strategies and negatively associated with providing support to the child. This may reflect a somewhat freeze response. Guilt has been shown to contribute to a maladaptive response to traumatic events (33). In the current study, feelings of guilt did not significantly contribute to the prediction of parental self-efficacy. Parents’ feelings of sadness as compared to guilt appeared to contribute to a more adaptive coping response, since they tended to be positively linked to providing child support and negatively linked to avoidance. These feelings may be closer to the mental pain experienced by the child after being bullied, thus enabling a more adaptive response. As predicted, anger was linked to retaliative coping. By contrast, and contrary to the hypothesis, worry contributed to child restrictions. This finding may suggest that when parents worry they are more likely to exercise control and restrictions, probably in an attempt to protect their child.

Overall, parents applied adaptive coping strategies (providing support to their child, turning to social support) more frequently than maladaptive coping strategies (retaliation, avoidance self-blame, inflicting child restrictions). Interestingly, retaliation, which is considered by professionals to be a maladaptive coping strategy because it escalates violence (34), reinforced parents’ sense of efficacy when dealing with bullying victimization. This was evident in one of the interviews conducted as part of the pilot for this study. A mother of a 12-year-old girl who had been victimized at school commented, “I was angry as hell and worried, worried for my child. I went to the school and talked to the teacher, but nothing helped until I went into the class and screamed at the kids that if anyone comes near my kid I don’t know what I’ll do to them. I didn’t threaten them that I’d beat them or anything, but they were scared … I was outraged, I threatened the principal that I would file a complaint with the Ministry of Education….” In the case of this mother and as shown in the general pattern of results, feelings of anger contributed to adopting a retaliative coping response. Some parents may resort to threatening or taking steps against the school or their child’s classmates/parents because it may be the only thing they believe will be effective. High levels of victimization were found to contribute to parents adopting a retaliative tactic; because bullying is a dynamic process, it might be that parents at first turned to more mellow coping strategies, but if the bullying intensified or continued over time, they escalated to retaliation. Ranson et al. (35) dubbed parents who act aggressively toward the school as “storming parents” and suggested that such parents are actually seeking mutual understanding from school administration when engaging in angry outbursts (34). However, these forms of parental retaliation can undermine trust and cooperation between the parents and the school staff. Thus, although retaliation contributed to parents’ sense of efficacy, it might not be what their child actually needs during the crisis. Retaliation was reported by only 27% of the parents. Nevertheless, this figure is important because these parents may need guidance and assistance.

Parents tended to seek more social support and information when they felt frustrated or offended by their child’s victimization. Surprisingly, seeking social support and information, which is considered an adaptive coping mechanism, was negatively associated with parents’ sense of efficacy in dealing with bullying. Thus, although social support can contribute to the construction of efficacy over time, within the short period of a given crisis, it may be perceived by parents as a sign of their inability to effectively deal with the situation on their own. Interestingly, social support was a fairly common coping strategy but might not be as empowering as expected, at least in the short term. Nonetheless, parents should be encouraged to keep seeking social support and information because it can contribute to parental efficacy over time by helping them to regulate negative emotions and providing information and valuable advice.

High level of child victimization was found to contribute to a maladaptive coping pattern among parents, in that child victimization level was predictive of parents adopting retaliative and avoidant coping and was negatively associated with child support. Parallel findings in the parenting literature suggest that the severity of a child’s problems may overwhelm parents emotionally such that they react less adaptively, which may undermine their sense of parental efficacy (36). For instance, Kuhn and Carter (37) showed that the severity of ADHD behaviors decreased mothers’ parental self-efficacy.

As hypothesized, providing support to the child as well as adopting a retaliation strategy were predictive of parental self-efficacy in dealing with victimization events. Parents’ feelings were not linked to parental self-efficacy above and beyond the coping strategies. Although the current study focused on the valence of emotions, the intensity of the emotion should also be taken into account in future studies as was suggested by Dix (36). Even though parents’ childhood history of victimization was found to decrease levels of concern regarding bullying in a previous study (21), in the current study it was not a significant contributing factor in parents’ coping or sense of efficacy. However, in the current study, this was measured by a single item, and more extensive qualitative and quantitative measures may be needed to better understand how parents’ past experiences with bullying might impact coping with their child’s victimization.

### Limitations

The current study has several limitations. Data were cross-sectional; thus, causality cannot be established. Future studies should include longitudinal designs so that parental self-efficacy can be assessed over time, which can provide a broader and more accurate picture of the mechanisms contributing to the establishment of parental efficacy in dealing with bullying victimization. Furthermore, the current study focused only on the parents’ perspective; it would be valuable in future studies to take into account the child’s experience of the parental coping response. An additional limitation is that parental self-efficacy in dealing with bullying was measured by a single item. Although a single item is considered a valid way to evaluate a general sense of self-esteem (38), future studies could benefit from including subscales probing different aspects of parental efficacy in dealing with bullying (27). For example, parents might have different perceptions of their efficacy in dealing effectively with their child as compared to peers or school policies. Furthermore, parents’ emotions were measured using self-reports, whereas emotions are multidimensional and include physiological changes in addition to the subjective experience reported in questionnaires. Future studies should include additional measures of emotions in order to better capture parents’ emotional experiences.

### Applications

The current findings have important bearing on antibullying prevention and intervention efforts. Making parents an integral part of interventions is vital to maximizing the effects of these interventions (for a systematic review, see 39, 40). However, most interventions that include parents only do so to a very limited extent, for example, by sending antibullying materials to parents, holding antibullying conferences, updating parents on school policies, or meeting individually with parents of children involved in bullying (41–43). Including parents to a greater extent in prevention and interventions efforts hinges on a better understanding of the psychological processes parents experience when dealing with bullying. The findings of the current study point to the complex emotions parents experience when their child is bullied. Thus, interventions should include emotion regulation training that can enable parents to recognize the different emotions they experience and learn to accept and regulate them so they can take the most productive steps forward for their child and their own well-being. In light of the findings here, it is especially important to face feelings of guilt and anger that may lead to maladaptive coping, while becoming aware of feelings of sadness, which were found to be adaptive. Interventions improving parents’ general emotional regulation abilities have been found to boost parental self-efficacy (44), suggesting that similar training in the context of antibullying interventions is important. In order to further enhance parents’ sense of efficacy, interventions need to provide information and guidance on the effectiveness of coping with the different dimensions of bullying. Special attention should be paid to parents who manifest aggressive reactions; professionals need to understand their position better and help them deal more constructively with bullying by utilizing prosocial coping strategies instead. Aiding parents to deal more effectively with their child’s victimization can help children gain valuable parental support during crisis of bullying victimization.

## Data Availability Statement

The datasets generated for this study are available on request to the corresponding author.

## Ethics Statement

This study was carried out in accordance with the recommendations of Department of Health and Human Services, Ministry of Health with written informed consent from all subjects. All subjects gave written informed consent in accordance with the Declaration of Helsinki. The protocol was approved by the Ethics Committee.

## Author Contributions

JB conducted the study and prepared all sections of the paper.

## Conflict of Interest

The author declares that the research was conducted in the absence of any commercial or financial relationships that could be construed as a potential conflict of interest.

## References

[1] Hospital, C.S. Mott Children’s 2017 “Mott poll report.” https://mottpoll.org/reports-surveys/bullying-and-internet-safety-are-top-health-concerns-parents Retrieved June 2018 from https://mottpoll.org/reports-surveys/bullying-and-internet-safety-are-top-health-concerns-parents.

[2] Moore SE Norman RE Suetani S Thomas HJ Sly PD Scott JG Consequences of bullying victimization in childhood and adolescence: a systematic review and meta-analysis. World J Psychiatry (2017) 7(1):60–76. 10.5498/wjp.v7.i1.60 28401049 PMC5371173

[3] Swearer SM Hymel S Understanding the psychology of bullying: moving toward a social-ecological diathesis–stress model. Am Psychol (2015) 70:344. 10.1037/a0038929 25961315

[4] Claes L Luyckx K Baetens I de Ven M Witteman C Bullying and victimization, depressive mood, and non-suicidal self-injury in adolescents: the moderating role of parental support. J Child Family Studies (2015) 24(11):3363–71. 10.1007/s10826-015-0138-2

[5] Harcourt S Green VA Bowden C It is everyone’s problem’: parents’ experiences of bullying.” N Z J Psychol (2015) 44:3.

[6] Sawyer JL Mishna F Pepler D Wiener J The missing voice: parents’ perspectives of bullying. Child Youth Serv Rev (2011) 33(10):1795–803. 10.1016/j.childyouth.2011.05.010

[7] Lazarus RS Folkman S Coping and adaptation. Handb Behav Med (1984) 282325: 208.

[8] Lazarus RS Stress and emotion: a new synthesis. New York: Springer Publishing Company (2006).

[9] Lazarus Richard S From psychological stress to the emotions: a history of changing outlooks. Ann Rev Psychol (1993) 44(1):1–22. 10.1146/annurev.ps.44.020193.000245 8434890

[10] Compas BE Connor-Smith JK Saltzman H Thomsen AH Wadsworth ME Coping with stress during childhood and adolescence: problems, progress, and potential in theory and research. Psychol Bull (2001) 127(1):87–127. 10.1037/0033-2909.127.1.87 11271757

[11] Visconti KJ Troop-Gordon W Prospective relations between children’s responses to peer victimization and their socioemotional adjustment. J Appl Dev Psychol (2010) 31(4):261–72. 10.1016/j.appdev.2010.05.003

[12] Hunter SC Boyle JME Appraisal and coping strategy use in victims of school bullying. Br J Educ Psychol (2004) 74:83–107. 10.1348/000709904322848833 15096300

[13] Hunter SC Borg MG The influence of emotional reaction on help seeking by victims of school bullying. Educ Psychol (2006) 26(6):813–26. 10.1080/01443410600941946

[14] Harcourt Jasperse M Green VA "“We were sad and we were angry”: A systematic review of parents’ perspectives on bullying." In Child & Youth Care Forum (2014) 43(3):373–91 Springer US. 10.1007/s10566-014-9243-4

[15] Smorti A Menesini E Smith PK Parents’ definitions of children’s bullying in a five-country comparison. J Cross-Cult Psychol (2003) 34(4):417–32. 10.1177/0022022103034004003

[16] Cassidy W Brown K Jackson M “Making kind cool”: Parents' suggestions for preventing cyber bullying and fostering cyber kindness. J Educ Comput Res (2012) 46(4):415–36. 10.2190/EC.46.4.f

[17] Kristensen SM Smith PK The use of coping strategies by danish children classed as bullies, victims, bully/victims, and not involved, in response to different (hypothetical) types of bullying. Scand J Psychol (2003) 44(5):479–88. 10.1046/j.1467-9450.2003.00369.x 15030114

[18] Smith PK Slee P Morita Y Catalano R Junger-Tas J Olweus D The nature of school bullying: a cross-national perspective. London: Psychology Press (1999).

[19] Eslea M Smith P K. Pupil and parent attitudes towards bullying in primary schools. Eur J Psychol Educ (2000) 15(2): 207–19. 10.1007/BF03173175

[20] Humphrey G Crisp B R. Bullying affects us too: Parental responses to bullying at kindergarten. Aust J Early Child (2008) 33(1):45–9. 10.1177/183693910803300108

[21] Cooper LA Nickerson A B. Parent retrospective recollections of bullying and current views, concerns, and strategies to cope with children’s bullying. Journal of child and family studies (2013) 22(4):526-540. 10.1007/s10826-012-9606-0

[22] Karga B-N Giaglis Parental views of children’s bullying experience, coping strategies and their association with parenting practices. In: School Bullying. (2013). Dekker & Kas, 2013 (Chapter 1). New-York: Nova Publishers.

[23] Jones TL Prinz RJ Potential roles of parental self-efficacy in parent and child adjustment: a review. Clin Psychol Rev (2005) 25(3):341–63. 10.1016/j.cpr.2004.12.004 15792853

[24] Bandura A The explanatory and predictive scope of self-efficacy theory. J Soc Clin Psychol (1986) 4(3):359–73. 10.1521/jscp.1986.4.3.359

[25] Chwalisz K Altmaier EM Russell DW Causal attributions, self-efficacy cognitions, and coping with stress. J Soc Clin Psychol (1992) 11(4):377–400. 10.1521/jscp.1992.11.4.377

[26] Melbourne, The Royal Children’s Hospital 2018 “Childhood bullying: how are parents coping?” Retrived June 2018 from https://www.rchpoll.org.au/polls/childhood-bullying-how-are-parents-coping/.

[27] Malm EK Henrich C Varjas K Meyers J Parental self-efficacy and bullying in elementary. J School Violence (2017) 16(4):411–25. 10.1080/15388220.2016.1168743

[28] WHO 2018. “NCDs | Global School-Based Student Health Survey (GSHS).” WHO. World Health Organization 2018 Retrieved June 2018 from: http://www.who.int/ncds/surveillance/gshs/en/.

[29] Kochenderfer-Ladd Becky Peer victimization: the role of emotions in adaptive and maladaptive coping. Soc Dev (2004) 13(3):329–49. 10.1111/j.1467-9507.2004.00271.x

[30] Kochenderfer-Ladd B Skinner K Children’s coping strategies: moderators of the effects of peer victimization?. Dev Psychol (2002) 38(2): 267. 11881761 10.1037//0012-1649.38.2.267

[31] Bandura A Guide for constructing self-efficacy scales. Self-Efficacy Beliefs Adolesc (2006) 5(1):307–37.

[32] Hale R Fox CL Murray M Hale R As a parent you become a tiger’: parents talking about bullying at school. J Child Family Studies (2017) 26(7):2000–15. 10.1007/s10826-017-0710-z PMC5487753 28680262

[33] Lee DA Scragg P Turner S The role of shame and guilt in traumatic events: a clinical model of shame-based and guilt-based PTSD. Br J Med Psychol (2001) 74(4):451–66. 10.1348/000711201161109 11780793

[34] Lovegrove PJ Bellmore AD Green JG Jens K Ostrov JM “My voice is not going to be silent”: What can parents do about children’s bullying? J Sch Violence (2013) 12(3):253–67. 10.1080/15388220.2013.792270

[35] Ranson S Martin J Vincent C Storming parents, schools and communicative inaction. Br J Sociol Educ (2004) 25(3):259–74. 10.1080/0142569042000216936

[36] Dix T The affective organization of parenting: adaptive and maladaptative processes. Psychol Bull (1991) 110(1):3. 10.1037//0033-2909.110.1.3 1891517

[37] Kuhn JC Carter AS Maternal self-efficacy and associated parenting cognitions among mothers of children with autism. Am J Orthopsychiatry (2006) 76(4):564–75. 10.1037/0002-9432.76.4.564 17209724

[38] Robins RW Hendin HM Trzesniewski KH Measuring global self-esteem: construct validation of a single-item measure and the Rosenberg Self-esteem Scale. Personal Soc Psychol Bull (2001) 27(2):151–61. 10.1177/0146167201272002

[39] Farrington DP Ttofi MM School-based programs to reduce bullying and victimization. Campbell Collab (2009) 6:1–149. 10.4073/csr.2009.6 PMC8356322 37131921

[40] Vreeman RC Carroll AE A systematic review of school-based interventions to prevent bullying. Arch Pediatr Adolesc Med (2007) 161(1):78–88. 10.1001/archpedi.161.1.78 17199071

[41] Sharp S Thompson D The role of whole-school policies in tackling bullying behaviour in schools. In: School Bullying: Insights and Perspectives. Routledge (1994). p. 57–83.

[42] Bonds M Stoker S Bully-proofing your school: a comprehensive approach for middle schools. London Sopris; West (2000).

[43] Sherer YC Nickerson AB Anti-bullying practices in American schools: perspectives of school psychologists. Psychol Schools (2010) 47(3):217–29. 10.1002/pits.20466

[44] Sanders MR Mazzucchelli TG The promotion of self-regulation through parenting interventions. Clin Child Family Psychol Rev (2013) 16(1):1–17. 10.1007/s10567-013-0129-z 23397366

